# Multispectral light scattering endoscopic imaging of esophageal precancer

**DOI:** 10.1038/lsa.2017.174

**Published:** 2018-04-06

**Authors:** Le Qiu, Ram Chuttani, Douglas K Pleskow, Vladimir Turzhitsky, Umar Khan, Yuri N Zakharov, Lei Zhang, Tyler M Berzin, Eric U Yee, Mandeep S Sawhney, Yunping Li, Edward Vitkin, Jeffrey D Goldsmith, Irving Itzkan, Lev T Perelman

**Affiliations:** 1 Center for Advanced Biomedical Imaging and Photonics, Division of Gastroenterology, Department of Medicine, Beth Israel Deaconess Medical Center, Harvard University, Boston, MA 02215, USA; 2 Division of Gastroenterology, Department of Medicine, Beth Israel Deaconess Medical Center, Harvard University, Boston, MA 02215, USA; 3 Department of Pathology, Beth Israel Deaconess Medical Center, Harvard University, Boston, MA 02215, USA; 4 Department of Anesthesia, Critical Care and Pain Medicine, Beth Israel Deaconess Medical Center, Harvard University, Boston, MA 02215, USA; 5 Biological and Biomedical Sciences Program, Harvard University, Boston, MA 02215, USA

**Keywords:** biophotonics, endoscopic multispectral imaging, light scattering spectroscopy, noninvasive cancer detection

## Abstract

Esophageal adenocarcinoma is the most rapidly growing cancer in America. Although the prognosis after diagnosis is unfavorable, the chance of a successful outcome increases tremendously if detected early while the lesion is still dysplastic. Unfortunately, the present standard-of-care, endoscopic surveillance, has major limitations, since dysplasia is invisible, often focal, and systematic biopsies typically sample less than one percent of the esophageal lining and therefore easily miss malignancies. To solve this problem we developed a multispectral light scattering endoscopic imaging system. It surveys the entire esophageal lining and accurately detects subcellular dysplastic changes. The system combines light scattering spectroscopy, which detects and identifies invisible dysplastic sites by analyzing light scattered from epithelial cells, with rapid scanning of the entire esophageal lining using a collimated broadband light beam delivered by an endoscopically compatible fiber optic probe. Here we report the results of the first comprehensive multispectral imaging study, conducted as part of routine endoscopic procedures performed on patients with suspected dysplasia. In a double-blind study that characterized the system’s ability to serve as a screening tool, 55 out of 57 patients were diagnosed correctly. In addition, a smaller double-blind comparison of the multispectral data in 24 patients with subsequent pathology at locations where 411 biopsies were collected yielded an accuracy of 90% in detecting individual locations of dysplasia, demonstrating the capability of this method to serve as a guide for biopsy.

## Introduction

Esophageal cancer is the fifth leading cause of cancer deaths worldwide^1^. The two primary types of esophageal cancer are adenocarcinoma and squamous cell carcinoma. The former is the leading esophageal malignancy in the Western world. Esophageal adenocarcinoma is also the malignancy that has been rising the fastest in the United States over the past four decades^2, 3^. It has very poor prognosis with less than 16% of patients alive five years after diagnosis. Worldwide, there are ~52 000 new esophageal adenocarcinoma cases every year^4^. Barrett’s esophagus (BE), a potentially precancerous condition which may arise in the setting of gastroesophageal reflux, precedes almost all cases of adenocarcinoma, with ~3 million Americans affected. Non-dysplastic Barrett’s (NDB) is the lowest risk form of Barrett’s esophagus, which generally progresses through the sequential stages of low (LGD) and high grade dysplasia (HGD) on the way to forming adenocarcinoma. There is widespread acceptance that Barrett’s esophagus with HGD should be treated endoscopically using various ablative therapies, which now have superb outcomes^5^. Some groups also advocate treating Barrett’s with LGD, although the rate of cancer progression in untreated LGD patients is still a matter of ongoing investigation^6^.

In order to initiate early, curative treatment, it is essential to recognize and diagnose high risk dysplastic changes in Barrett’s epithelium. HGD, the critical step before invasive esophageal adenocarcinoma^7^, is characterized primarily by alterations in cellular and glandular architecture, such as nuclear enlargement, crowding, stratification, loss of nuclear polarity, hyperchromatism, and cribriform growth^8^. Surveillance for HGD uses standard optical endoscopy with a prescribed pattern of biopsy sampling. This procedure, when a small fraction of BE tissue is examined by pathology, has a relatively low probability of detecting dysplastic or early cancerous changes, due to the usually macroscopically invisible nature of early, high risk lesions. This standard of care is flawed. Recent meta-analysis of 24 studies determined that 25% of esophageal adenocarcinomas are diagnosed within one year of an endoscopic surveillance of Barrett’s patients during which no HGD was detected^9^.

Several imaging approaches have been explored recently to visualize dysplasia in BE. These technologies include: autofluorescence imaging (AFI) combined with high resolution endoscopy (HRE) and narrow band imaging (NBI)^10^, chromoendoscopy^11^, confocal laser endomicroscopy (CLE)^12, 13^, optical coherence tomography (OCT)^14, 15, 16^, angle-resolved low-coherence interferometry (a/LCI)^17^, and photoacoustic endoscopy (PAE)^18^. In addition, spectroscopy based techniques, such as laser-induced fluorescence spectroscopy (LIF)^19^, elastic scattering spectroscopy (ESS)^20, 21^, partial wave spectroscopy (PWS)^22^ and diffuse reflectance spectroscopy (DRS)^23^ have also been investigated. Although these techniques show promise, no technique has yet achieved the performance required for clinical acceptance.

The main stumbling block in visualizing dysplasia in BE is the need for a single technique to simultaneously achieve subcellular scale sensitivity and anatomic scale imaging. To overcome this problem we developed a multispectral imaging system that combines light scattering spectroscopy (LSS), which analyzes light scattered from epithelial cells, thereby identifying otherwise invisible dysplastic sites^24^, with rapid scanning of the entire esophageal lining by a collimated broadband light beam, delivered by an endoscopically compatible fiber optic probe^25^. The system scans the entire Barrett's esophagus segment in minutes, providing the endoscopist with real time information about the location of invisible HGD. Here we report the results of the first comprehensive multispectral imaging study in 57 patients with suspected dysplasia while they were undergoing routine endoscopy, thereby evaluating the capability of our method to serve as a screening tool and a guide for biopsy.

## Materials and methods

### Light scattering spectroscopy

Human tissue is optically turbid which means elastic scattering dominates light transport. Tissue primary scattering centers are the extracellular matrix consisting of a collagen fiber network and intracellular structures with sizes smaller than optical wavelengths^26^. Larger intracellular structures, such as nuclei, also scatter light, with their relative contribution increasing in the backscatter direction. Enlarged, crowded and dense epithelial cell nuclei are the primary histological features of dysplasia and cancer in BE^27^. Thus, a technique which connects the spectroscopic characteristics of light scattered elastically by the epithelium to epithelial cell morphology can be used for BE dysplasia diagnosis. Such a technique is called biomedical light scattering spectroscopy, or LSS^24^.

**Figure 1:**
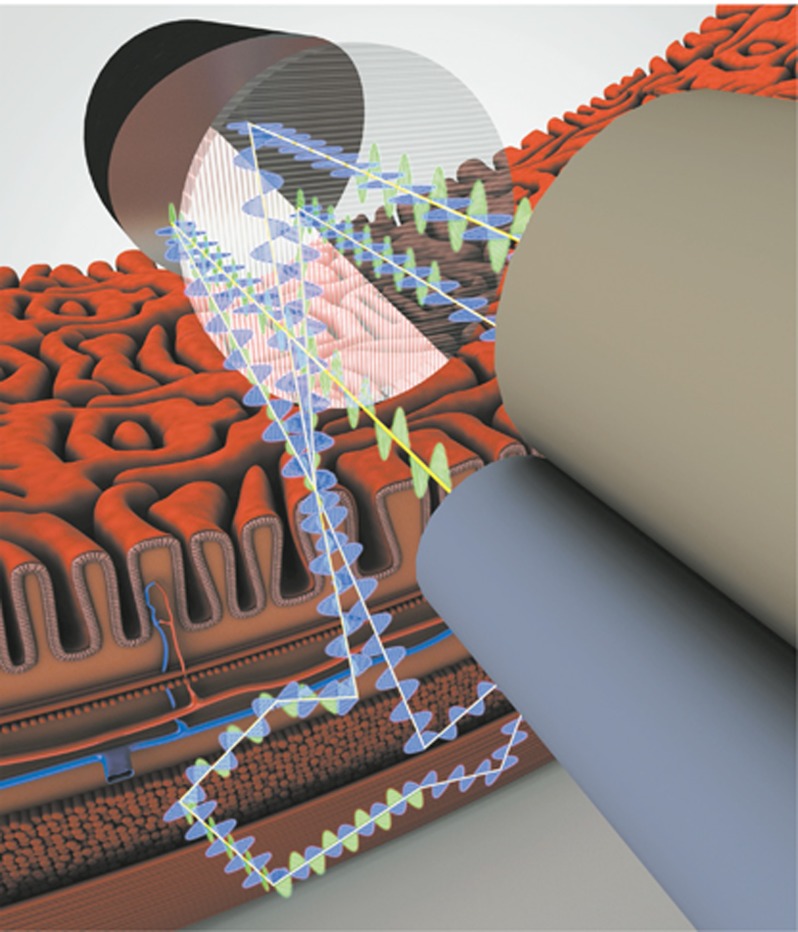
Polarization gating light scattering spectroscopy. Barrett's esophagus tissue is illuminated with polarized light emitted from the polarized scanning fiber optic probe. The backscattered light from the cells in the superficial layer of columnar epithelium is polarized parallel to the incoming light, while the light reflected from the deeper tissues becomes depolarized, containing equal amounts of parallel and perpendicular polarizations. Subtracting the two polarizations cancels out the contribution of deeper tissues and the resulting signal is proportional to the signal from the epithelium, which contains the information about early precancerous changes.

In LSS, it is not possible to measure single backscattering events directly in human tissue. As light propagates in tissue, multiple scattering randomizes information about the scatterers over the effective scattering length. Fortunately, within the thin epithelial layer at the surface the light is not randomized completely and it is in this layer where malignancy begins. In this region the elastic scattering process is preserved. Thus, one can divide the light reflected from the tissue’s epithelial lining into two components: single backscattering from cell nuclei in the upper tissue structure, and a diffusely scattered background. In order to extract the single scattering component, which contains the essential information, the diffusive background can be removed using either polarization^25, 28, 29, 30^ or spatial^31^ gating approaches. The light scattered back from the epithelial cells located at the surface retains the incoming polarization, while diffuse scattering from the deeper tissue regions layers is depolarized (see Figure 1). Subtracting parallel and perpendicular signals cancels the deeper tissue effect. The result is then related to the contribution of the epithelial cells, which is analyzed to provide the information about early precancerous changes.

In our *in vivo* imaging study in 57 patients described in the Results and discussion, we use the polarization gating LSS technique described above, employing the multispectral light scattering endoscopic imaging system described in the following section.

### Multispectral light scattering endoscopic imaging system

**Figure 2:**
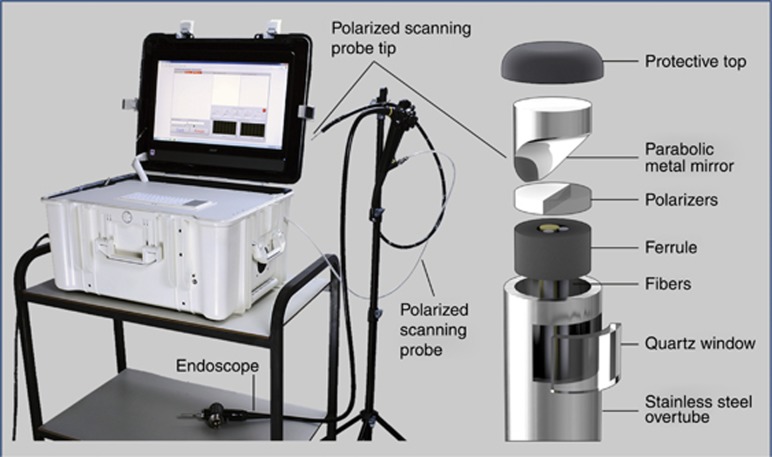
Endoscopic multispectral scanning imaging system. The photograph on the left shows the system on a cart with its scanning probe inserted into the working channel of an Olympus GIF-H180 endoscope. The schematic on the right shows the exploded view of the polarized scanning probe tip. When assembled the parabolic mirror is opposite the quartz window.

The system, fitting in a carry-on case is shown in Figure 2. A multichannel spectrometer, broadband bright LED light source, and power supply are installed in the bottom of the case. A computer for system control is also located in the bottom of the case and is used for data analysis and visualization of the diagnostic information. The middle shelf is made of transparent polycarbonate and houses the probe control box and three fiber connectors for light delivery one of which is connected to the light source, and parallel and perpendicular polarization fibers connected to two spectrometer channels. This easily accessible shelf is also used for safe storage of the probe before the clinical procedure or when the system is moved between clinical sites. A hinged door installed on the side wall of the case allows convenient extension of the probe from the case permitting it to be inserted in the working channel of a commercial endoscope, without the need to disconnect and reconnect the probe. The upper shelf of the system has a built-in keyboard, mouse and a slot for probe calibration that gives the probe access to a small light-tight enclosure housing with an attached Spectralon reflectance standard. The cover of the case houses a flat touch screen.

The system is designed to maximize the scanning speed, thereby ensuring that the duration of the endoscopy procedure would not be increased by more than several minutes. It uses a scanning polarization gated fiber probe that is inserted into a standard gastroendoscope working channel during endoscopy. Light from a broadband high brightness LED light source is coupled into a 400-μm core delivery fiber (NA=0.22) which is attached to a 200 μm thick, 1.5-mm diameter cylindrical linear polarizer/analyzer with two orthogonal polarization components. The delivery fiber is adjacent to two 200-μm core collection fibers (NA=0.22), each behind a linear polarizer which is shaped to ensure that one fiber collects light with the same polarization as the delivery fiber and the other collects perpendicularly polarized light. It is necessary to mount the polarizers at the distal tip since multimode fibers do not maintain polarization. At the probe distal tip a parabolic mirror (Figure 2, right panel) collimates the illumination beam and collects light from the 2-mm illuminated spot. This compensates for peristaltic and other motions of the esophagus. Collimation ensures that the spectrum is not affected by distance or orientation. The majority of the procedures are performed with gentle air insufflation of the esophagus, maintaining a relatively stable tubular shape and preventing the optical probe from touching the esophageal walls. However, even if the probe comes in contact with the wall, the overlap of the illumination and collection fields at the probe window is 75% which is adequate for evaluating the backscattering component. With the increase of the distance the overlap is also increasing and becomes optimal at 11 mm distance from the axis of the probe, although the degree of overlap remains adequate up to ~20 mm from the probe axis. To avoid specular reflection, light is directed ~70° to the axis of the probe. The probe is protected by the parylene coated stainless steel torque tube connected to two stepper motors of the control box, providing rotary and axial scanning. To increase scanning accuracy the data collection is performed only during the counterclockwise rotational scan. After the data collection is complete, the probe rotates 1.25 revolutions clockwise and then 0.25 revolution counterclockwise to minimize hysteresis of the torque tube and ensure the same angular position for the start of the next rotational scan.

To be able to collect the data simultaneosly with NBI illumination, which employs three illumination filters centered at 415, 445, and 500 nm, and also to minimize the interference from the hemoglobin Soret band (400–440 nm) and Q bands (500–600 nm) we analyzed data in the 600 to 800 nm spectral range. This 200-nm wide spectral band is sufficient to capture LSS features needed for epithelium evaluation.

A computer running a software interface written in LabVIEW operates the control box and performs data collection. The commands of the control box are synchronized with scanning of the illumination fibers and spectrometer data capture. Video acquired from the camera of the endoscope is used in conjunction with the pseudo-color map presentation of the LSS data. Each 30 data point rotary scan is followed by a 2-mm axial step. The system collects 300 data points for each 2-cm segment of Barrett’s esophagus in less than one minute, including the repositioning time.

### Diagnostic algorithm

The backscattering spectrum collected with the polarized scanning probe at each locally illuminated spot is found by subtracting experimentally measured perpendicularly polarized reflectance spectrum from a parallel polarized one, 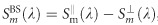, and by normalizing it to remove peristalsis-related amplitude variations (see Supplementary Fig. S1). It can be represented as a sum of the backscattered spectra of the subcellular organelles present in the illuminated spot


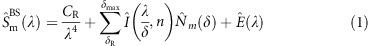


where *δ* is the organelle size, *n* is its refractive index, 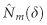 is the organelles’ discreet size distribution, 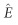 is the experimental noise, 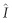 is the single scatterer Mie spectrum, *λ* is the wavelength, and *m* represents spatial location. Here the first term *C*_R_/*λ*^4^ accounts for effects of Rayleigh scattering due to macromolecules and other scatterers smaller than *δ*_R_=100 nm, with *C*_R_~*Nδ*^6^/*r*^2^ where *N* is the number of Rayleigh scatterers, *r* is the distance from the scatterers to the detector^32^, and *δ*_max_ is the maximum biologically realistic nuclear size considered in the calculations. In order to extract the organelles’ size distributions, Equation (1) can be solved using a linear least squares algorithm with non-negativity constraints^33^ with methods described elsewhere^34, 35^. With this algorithm we accurately reconstruct the epithelial cell nuclei size distribution at the illuminated spot.

For the system to be useful, it is necessary to distinguish HGD and LGD sites from NDB in the clinic in near real time during endoscopy. To this end, we developed a rapid semi-empirical algorithm, based on the above physical considerations. In this algorithm, instead of the lengthy process of reconstructing the size distributions of the nuclei present in each locally illuminated spot, a simple diagnostic parameter Δ_*m*_ is calculated for each measurement site *m*. To accomplish this, the difference of the normalized backscattering spectrum 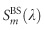 and the normalized root mean square (RMS) spectrum 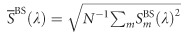 is computed, where *N* is the number of the measurement sites, providing a diagnostic parameter Δ_*m*_ for each site


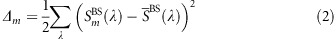


where the summation is performed over a discrete set of wavelengths representing the spectrum. The diagnostic parameter Δ_*m*_ is higher for dysplastic sites because the normalized RMS spectrum 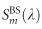 is dominated by the spectra of the normal sites and therefore has the same shape as the spectrum of a normal site. As a result, for normal sites, Δ_*m*_ calculated using Equation (2) is very small. However, for the dysplastic sites the spectrum is considerably different because of the contribution from the larger dysplastic nuclei. As a result, the spectra for dysplastic sites 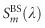 are sufficiently different from 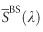, resulting in a much larger Δ_*m*_. Therefore Δ_*m*_ is a measure of the contribution from dysplastic cells.

The nuclear size distributions were reconstructed from the LSS data for various spatial locations. From these distributions we determined that a site should be considered dysplastic if the diagnostic parameter exceeds 0.1. This Δ=0.1 threshold is equivalent to ~25% contribution from enlarged nuclei, defined as nuclei over 9 microns in diameter^25^. To verify this cut-off we now performed an ROC analysis on the complete data set (see Supplementary Fig. S2) and determined that Δ=0.1 is indeed very close to the optimal cut-off. This straightforward diagnostic rule allows the analysis to be performed in real time.

**Figure 3:**
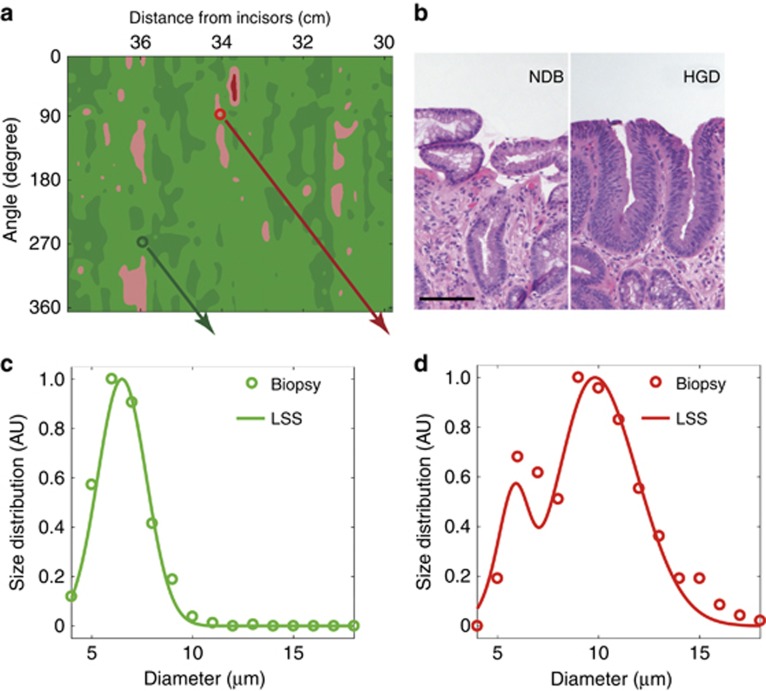
Nuclear size distributions for one dysplastic and one non-dysplastic sites in Barrett’s esophagus. (**a**) Red and pink regions of the map indicate areas suspicious for dysplasia based on nuclear size distributions extracted from the backscattering spectra for each individual spatial location, with Δ below 0.05 colored dark green, 0.05–0.10 colored light green, 0.10–0.15 colored pink, and above 0.15 as red. The circles indicate the locations of two biopsies histologically diagnosed as non-dysplastic biopsy (NDB) and high-grade dysplasia (HGD), and marked with green and red circles, respectively. (**b**) Histology images from biopsies collected in the marked locations, with NDB on the left and HGD on the right (scale bar is 100 μm). Comparison of the nuclear size distribution obtained from the quantitative morphometric measurements (circles) from biopsies presented in panel b and reconstructed from the *in vivo* LSS data (solid lines) collected at the same NDB (**c**) and HGD (**d**) locations.

Typical pseudo-color map indicating areas suspicious for dysplasia for one of the subjects is shown in Figure 3. We also show the histology images from biopsies collected during the procedure for two of the locations marked on this map, with one of the locations later diagnosed as NDB and another as HGD. Comparison of the nuclear size distribution obtained from the quantitative morphometric measurements from those histology images and extracted from the backscattering LSS spectra collected *in vivo* show excellent agreement (Figure 3c and 3d), with non-dysplastic Barrett’s esophagus site having nuclear size distribution centered about 5 to 7 μm diameter while site marked as suspicious for dysplasia having nuclear size distributions with a main peak centered from 9 to 15 μm. These pseudo-color maps were presented to the endoscopist in cases where the endoscopic multispectral system was employed to guide biopsy. This permits the endoscopist to collect confirmatory biopsies at suspicious sites, minimizing the number of biopsies collected at non-dysplastic sites, reducing the labor, and shortening the screening and diagnostic times. It also causes less patient discomfort, ensures reliable detection of all precancerous lesions and in the future, should lead to immediate, focused treatment during a single endoscopy.

### Biopsy guidance

Currently the implemented guided biopsy procedure provides the gastroenterologist with the azimuthal coordinate and the distance from the upper incisors associated with the suspected dysplastic site. In addition, the system user interface displays a pseudo-color map highlighting suspected dysplastic areas and a freeze-frame endoscopy image, which shows the LSS spot where the data of a particular suspicious location was acquired. There are many landmarks in the BE that are observable under NBI. Using this information, the endoscopist can address the same spot within a distance of 5 mm.

In addition to the currently implemented procedure, and in order to simplify biopsy guidance and improve its accuracy, we developed a method for simultaneous and continuous visualizing suspected dysplasia sites. Since the scanning fiber probe and biopsy forceps both utilize the same accessory channel of an endoscope, they cannot be used simultaneously to guide biopsy. Cautery marking has recently been demonstrated as an approach for dealing with the problem of a single accessory channel and was used in a pilot clinical study^36^. Here, a balloon catheter was used to stabilize the esophagus and a cautery mark was made with a laser while imaging and targeting the suspicious region. This approach requires several seconds of stability while the mark is being placed. Our system does not use a balloon catheter to stabilize the esophagus, thus, if used in our system, cauterization marking would lose accuracy due to peristaltic motion. On the other hand, virtual marks can be more accurate than cauterization, can be implemented automatically in the software interface, and can make the procedure and clinical workflow of guided biopsy significantly shorter and smoother.

**Figure 4:**
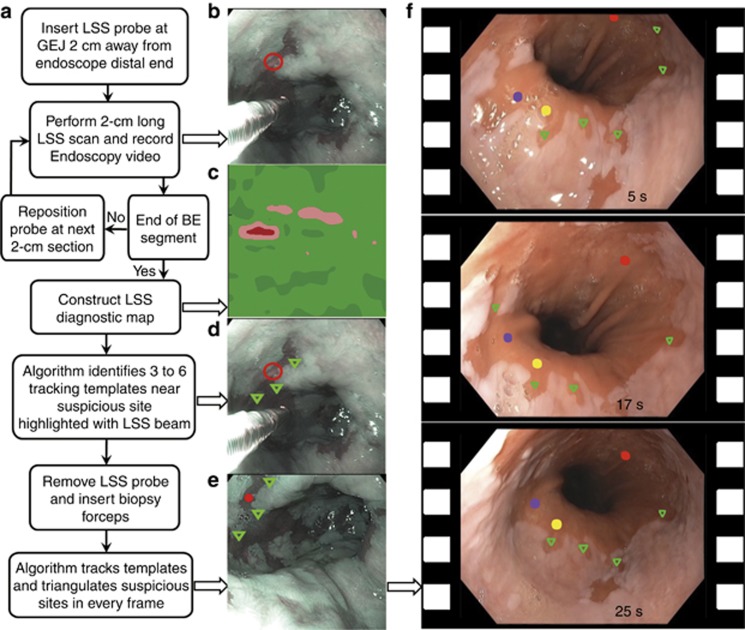
Biopsy guidance with feature tracking based virtual marks. (**a**) Flow chart of the biopsy guidance algorithm. (**b**) Video frame with location suspicious for HGD highlighted with the LSS beam and marked with red empty circle. (**c**) LSS diagnostic map of the corresponding 2-cm long BE section. (**d**) Same video frame as in B but with three trackable features identified by the algorithm and indicated with green triangles. Three to six trackable features are identified on a frame with a location suspicious for HGD. (**e**) Location suspicious for HGD is triangulated and tracked on every consecutive video frame (red solid circle). (**f**) Examples of the video frames with three locations suspicious for HGD (marked with red, yellow, and violet solid circles) and four to seven trackable features per frame (green triangles). GEJ—gastroesophageal junction.

Therefore, we designed a virtual marking algorithm that automatically identifies regions of interest during the spectroscopic scan and locates them later, during biopsy, whenever the same region again comes into view. While the dysplastic sites are usually visually indistinguishable, the border between normal squamous mucosa and Barrett's columnar mucosa, called the squamocolumnar junction is unique, possessing clearly identifiable features such as columnar mucosal tongues and islands. In cases when the squamocolumnar junction is not visible in the same frame as the site suspicious for dysplasia, the algorithm defines several highest intensity-gradient areas in the frame as the trackable features. We employed the Color-Ciratefi^37^ based process of template matching, comparing automatically chosen and stored template images, containing trackable features, with every incoming video frame, testing to identify the regions with highest similarity (Figure 4). Although the Color-Ciratefi is not the fastest template matching technique, it is ideally suited for effective parallelization.

The current algorithm implementation was tested using the on-board computer of the portable system. Supplementary Movie S1 illustrates tracking of several locations suspicious for dysplasia with suspicious locations as well as trackable features shown. The algorithm is not yet parallelized, and therefore is presently not fast enough for real-time biopsy guidance. However, with the addition of a graphics processing unit (GPU) computing processor to the endoscopic multispectral scanning imaging system, and using the algorithm in a parallel configuration, adequate processing speed should be achieved in the near future for real-time virtual marking based guidance.

## Results and discussion

We enrolled 57 patients at the Theodore and Cynthia Berenson Center for Advanced Endoscopy, a high-volume referral center for Barrett’s esophagus treatment. Patients who were referred for evaluation had all undergone prior endoscopies by other gastroenterologists, and had been referred because biopsy had confirmed BE with suspicion for dysplasia. The protocol was reviewed and approved by the Institutional Review Board.

**Figure 5:**
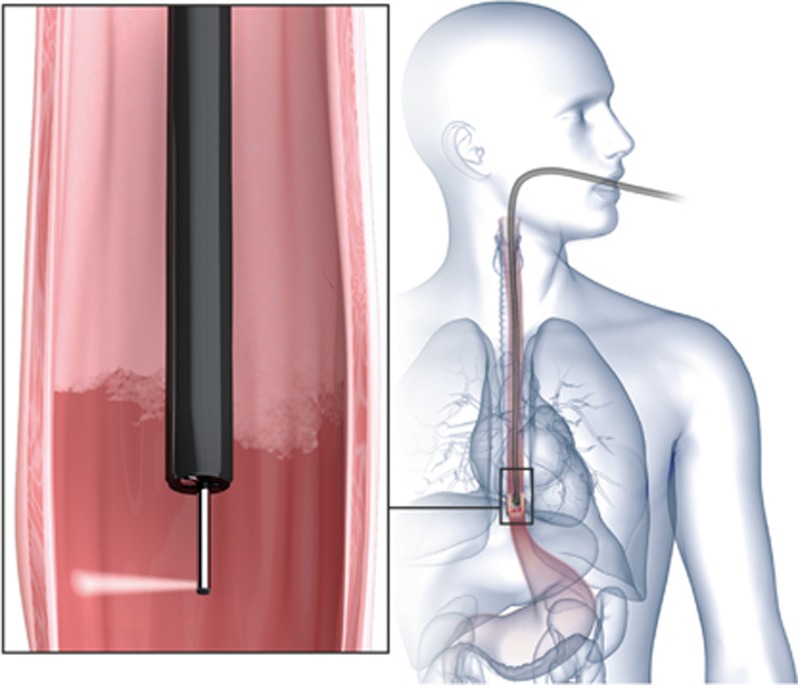
Clinical procedure with endoscopic multispectral scanning imaging system. The clinical system contains a probe that is inserted into the endoscope accessory channel. The fiber probe performs rapid automated rotational/longitudinal scanning of the entire BE segment.

During endoscopy, the probe was inserted into the Olympus GIF-H180 gastroscope working channel and extended two centimeters beyond the gastroscope tip to the distal edge of the BE segment (Figure 5). Each scan consisted of the automated rotary and linear backward withdrawal motion covering the entire two centimeters segment length. The endoscope tip was then withdrawn two centimeters and the next segment of BE was automatically examined.

### Performance evaluation

The method capabilities were validated by comparing the LSS results with the pathology results obtained subsequently at each location where biopsies were collected. The biopsied locations were determined by the distances from the incisors and from the angles of the probe relative to the initial position of the system scan, and recorded. The diagnostic results were presented in a form of pseudocolor LSS maps. Pathological examination of 872 collected biopsies revealed a total of 114 dysplastic sites. The remaining sites were diagnosed as non-dysplastic.

System performance was evaluated in two separate double-blind studies, emphasizing (1) biopsy based diagnosis and (2) patient based diagnosis. The former approach, which characterizes the system potential for performing guided biopsy, is based on a comparison of the LSS maps with the pathology reports in a double-blind fashion, and identifying true-positive, true-negative, false-positive, and false-negative sites for every biopsy location. The latter approach, which characterizes the system ability to serve as a screening tool, emphasizes identifying the presence of dysplasia in a particular BE patient rather than localizing the individual dysplastic sites. The rationale for this approach is that at the present time, once biopsies are diagnosed as HGD, radiofrequency ablation (RFA) and cryoablation therapies are applied to the entire BE segment, rather than the specific sites where HGD biopsies were collected^5, 38^.

**Figure 6:**
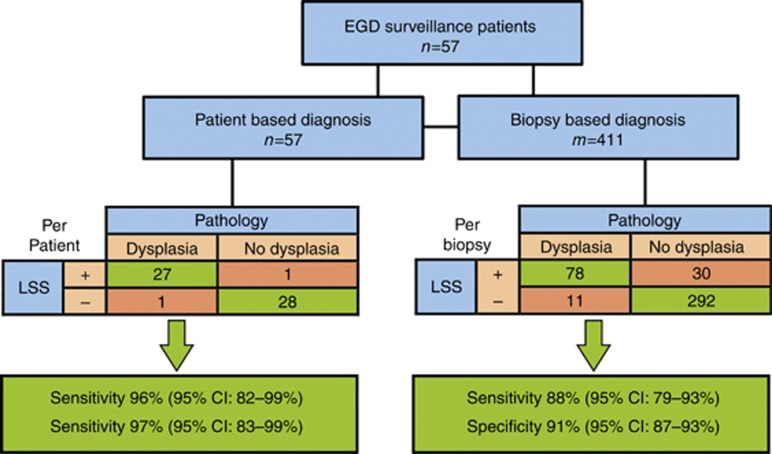
Diagnostic flow chart. Diagnostic flow chart of endoscopic multispectral scanning imaging system performance in BE patients. EGD indicates esophagogastroduodenoscopy.

The performance of the system in both studies is outlined in Figure 6. In the patient based diagnosis study in 57 subjects the double-blind comparison of LSS data with pathology reports revealed 27 true-positive cases, 1 false-positive case, 28 true-negative cases and 1 false-negative case (Figure 6). Overall, 55 out of 57 patients were diagnosed correctly in this study. Thus, LSS measurements in the patient based diagnosis study are characterized by a sensitivity of 96% with a 95% confidence interval (CI) of 82–99% and a specificity of 97% (95% CI: 83–99%).

**Figure 7:**
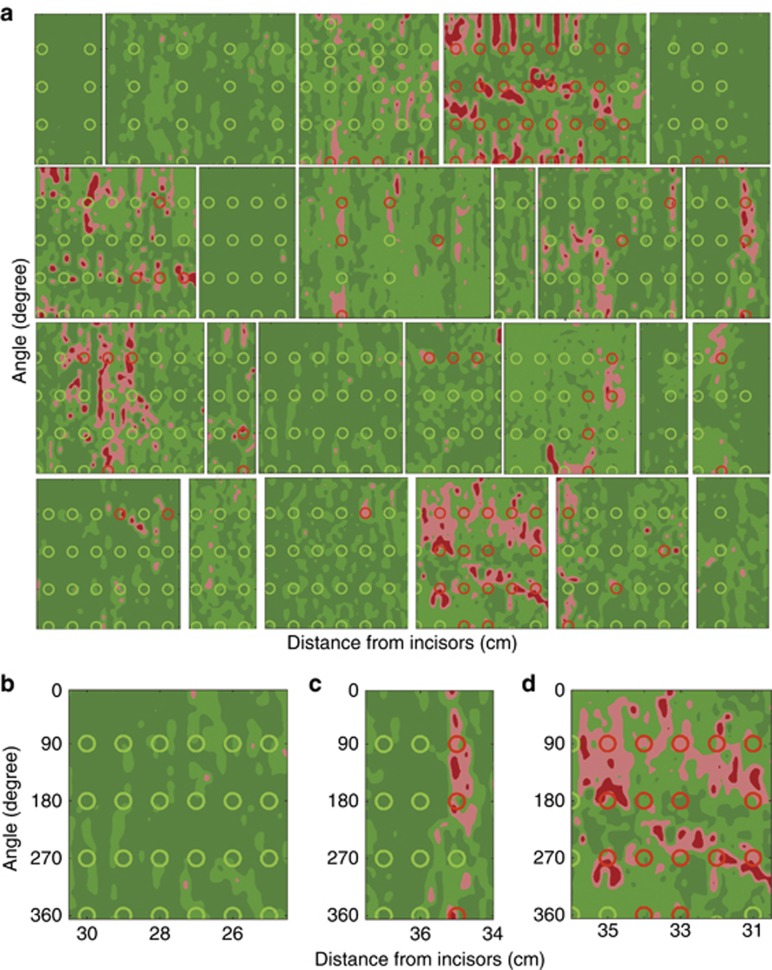
Pseudo-color maps highlighting areas suspicious for dysplasia in 24 subjects. (**a**) Maps produced from LSS data are overlaid with circles indicating biopsy sites and confirmed pathology. The vertical direction indicates the angle of rotation from the start of each rotary scan; the horizontal direction indicates the distance from upper incisors. Green map areas of various shades represent epithelium unlikely for HGD and red and pink map areas represent areas suspicious for HGD, as determined by LSS. Red and green circles indicate biopsy sites of HGD and non-dysplastic Barrett’s esophagus, respectively, as determined by pathology. Maps (**b**–**d**) are typical maps and biopsies for subjects with no areas suspicious for HGD, focal HGD suspicious areas, and significant HGD suspicious areas, respectively.

A smaller biopsy based diagnosis study in 24 subjects was performed to evaluate potential accuracy of multispectral system biopsy guidance (Figure 7). Here double-blind comparison of LSS data with biopsy reports yielded an accuracy of 90% (95% CI: 87–93%) with a sensitivity of 88% (95% CI: 79–93%) and a specificity of 91% (95% CI: 87–93%) in detecting individual locations of HGD (see Figure 4) with the AUC of the ROC being 0.95 (95% CI: 93–97%). This means that when dysplasia is present in BE, the LSS guided biopsy procedure has a 99.5% probability to locate it with just 4 guided biopsies, while the standard-of-care Seattle protocol has ~43% chance of detection with 20 biopsies^39^.

Both sensitivity and specificity of the per-patient study are improved compared to the per-biopsy study. The reason for the improvement of sensitivity is evident since the probability of finding at least one true positive increases with each additional measurement. The improvement in specificity is less evident and arises from the fact that almost all false positives are found in subjects with dysplasia, and not included in the per-patient specificity calculation. This is because the bias introduced in the mean spectra due to a large number of dysplastic sites in patients with dysplasia compared with patients with no dysplasia is larger (see Equation (2)), increasing the false positive rate.

In several enrolled BE patients, pathology revealed no dysplasia while the LSS scans indicated sites suspicious for dysplasia, in locations where no biopsies had been collected. These foci of dysplasia were missed by the standard-of-care procedure, which blindly biopsies a tiny fraction of the esophageal tissue using a prescribed systematic pattern, but were caught by the multispectral system. In follow-up procedures, guided biopsies were performed at the suspicious sites, each time revealing high grade dysplasia. These patients were given RFA treatment, in all likelihood preventing the development of invasive adenocarcinoma, and probably saving their lives. We conclude that the endoscopic multispectral scanning imaging system offers great promise for the ability to detect dysplasia in BE.

### Discussion

Presently, high resolution endoscopy (HRE)^10, 40, 41^ in combination with narrow band imaging (NBI) and similar hemoglobin absorption enhancement techniques^42, 43^, are now provided by all three leading endoscope manufacturers, Olympus, Pentax and Fujinon. This approach still misses at least a quarter of the dysplastic areas in Barrett’s esophagus which are at high risk for developing adenocarcinomas, in a time interval often less than one year in certain patients^9^. Therefore, several new optical approaches have been developed recently, commercialized and approved for clinical use, either in the US or in Europe, in order to achieve the performance required for a new biopsy guidance technique to replace or supplement the current standard of care. The American Society for Gastrointestinal Endoscopy (ASGE)^44^ specifies that such a technique should have a 90% per-patient sensitivity, 80% specificity, and 98% or greater negative predictive value (NPV) for detecting HGD.

One of these approaches is autofluorescence imaging (AFI)^45^ endoscopy, marketed by Olympus in Europe and Asia. The AFI endoscopy instruments target the strongest native tissue fluorophores, collagen and elastin, located in the submucosal layer of the esophagus. Since dysplastic changes are mainly associated with the epithelial morphology and dysplastic epithelium does not feature any prominent fluorophores, the assumption here is that autofluorescent radiation generated in the submucosa will be attenuated differently when passing through the epithelium, highlighting dysplastic changes. Recent studies demonstrated that while AFI can improve the detection rate of HGD in BE when used by an experienced operator^46^, it also has a relatively high false-positive rate (81%)^47^. Therefore, AFI endoscopy could be useful as a rapid initial evaluation technique to identify suspicious locations in BE, but would require subsequent assessment with another more specific technique for HGD confirmation.

Another promising technology is optical coherence tomography (OCT), a cross-sectional optical imaging technique analogous to ultrasound^48^. The resolution of OCT is approximately tenfold greater than the resolution of a high frequency endoscopic ultrasound approaching that of light microscopy. Feasibility of the endoscopic OCT to identify precancer and early cancer in BE with various research OCT instruments has shown considerable promise^14, 15, 49, 50^, with one study in 55 patients demonstrating a sensitivity of 83% and specificity of 75% for combined diagnoses of intramucosal carcinoma (IMC) and HGD in BE^51^ and another study in 33 patients demonstrating a sensitivity of 68% and specificity of 82% for detecting HGD in BE^52^. Upper endoscopy compatible OCT system for BE evaluation, NvisionVLE, has recently been commercialized by NinePoint Medical. Extensive sensitivity and specificity data for HGD detection in BE with the NvisionVLE system in a large study population has yet to be published. The main advantages of OCT are high speed, the ability to image a mucosal layered structure of BE in real time, and a substantial probing depth (several millimeters). Its limitations are the variability in image interpretation and the lack of subcellular resolution.

Another upper endoscopy compatible optical technology is confocal laser endomicroscopy (CLE). Here the gastroenterologist obtains high resolution microscopic images of epithelial cells to depths up to 130 μm in near real-time, with a field of view limited to a few hundred microns. Currently two upper endoscopy compatible CLE systems are commercially available, a reflectance confocal probe-based Cellvizio system from Mauna Kea Technologies and a fluorescence endoscope-integrated Optiscan confocal microscope from Pentax^53^. Improved sources of contrast, such as two-photon fluorescence (TPF) have also been recently suggested^54, 55^. Several studies in BE patients^55^ demonstrated more than 80% accuracy for diagnosing dysplasia when preceded by high-definition white-light endoscopy (HD-WLE) as an initial targeting approach. A recent meta-analysis study summarized the CLE diagnostic results of eight studies. Here, 709 patients and 4008 specimens showed a sensitivity of 70% and specificity of 91% for per biopsy evaluation and sensitivity of 89% and specificity of 75% for per patient analysis in detecting neoplasia^56^. Another meta-analysis of 7 studies in 373 patients and 3493 specimens reported a sensitivity of 58% and a specificity of 90% per biopsy and a sensitivity of 79% and specificity of 90% per patient for another set of 4 studies in 346 subjects and 2304 specimens^57^. The outcomes appeared to depend on the operator’s experience^58, 59, 60^. To summarize, the main advantage of CLE is its ability to produce histology like images, while limitations are related to the small field of view, making screening of the entire esophagus impractical.

Table 1 compares recently commercialized optical technologies which offer biopsy guidance for detecting dysplasia in BE with LSS endoscopic imaging presented here, providing information on the performance of each technique. All these technologies rely on the endoscopist’s ability to recognize dysplastic features in the images provided, and therefore are dependent on the operator’s training and experience. They also require real-time human processing of a rapid video stream, which is often challenging. On the other hand, the endoscopic multispectral scanning imaging technique discussed in this paper does not suffer from inter-observer or intra-observer variability and interpretation, as it requires no operator training or human processing. It rapidly surveys the entire Barrett's segment while at the same time retaining subcellular sensitivity, accurately locating dysplasia in tissue with no visible abnormalities when observed with white light or fluorescence.

## Conclusions

In the per-patient evaluation study, the multispectral light scattering endoscopic imaging system demonstrated a sensitivity of 96% and a specificity of 97% for high grade dysplasia detection, exceeding the respective characteristics required by the ASGE^43^. In our study, the prevalence adjusted^61^ negative predictive value (NPV), a critically important characteristic for an effective screening test, is 99.5%, which is also exceeds the required 98%. In the per-biopsy evaluation study, a specificity of 91%, a sensitivity of 88%, and an NPV of 96% in detecting individual locations of HGD were demonstrated. It is important to emphasize here that since commonly performed RFA therapy is applied to the entire BE segment, per-patient evaluation should be sufficient.

In conclusion, we demonstrated the capability of the multispectral light scattering endoscopic imaging to accurately detect esophageal precancer. By using macroscopic spectral measurements to analyze microscopic subcellular structure, the multispectral technique described herein locates dysplastic esophageal tissue without any visual cues. It provides rapid endoscopically compatible scanning of the entire esophageal surface within minutes, requires no external contrast agents, and is very safe.

## Figures and Tables

**Table 1:** LSS endoscopic imaging performance comparison vs. recently commercialized optical technologies to provide biopsy guidance for detecting dysplasia in BE

Technique	Method	Sites	Patients	Sensitivity %	Specificity %	Operator Independent	Entire Surface	Ref
AFI	B	74	63	56/75**	78/76**		√	^41^
AFI+HRE	B	74	63	64/84**	83/85**			^41^
OCT	B	177	55	83	75		√	^46^
	B	314	33	68	82			^47^
CLE	B/P	4008*	709	70/89	91/75			^51^
	B/P	3493*	346	58/79	90/90			^52^
LSS	B/P	411	57	88/96	91/97	√	√	This study

B—per-biopsy evaluation; P—per-patient evaluation; AFI—autofluorescence imaging; AFI+HRE− autofluorescence imaging combined with high resolution endoscopy; OCT—optical coherence tomography; CLE—confocal laser endomicroscopy; LSS—light scattering spectroscopy; *meta-analysis; **non-experts/experts.
