# Supplementary material for: Multispectral light scattering endoscopic imaging of esophageal precancer
Source: Light Sci Appl. 2018 Apr 6;7:17174–. doi: 10.1038/lsa.2017.174 (PMC6060057; doi:10.1038/lsa.2017.174)
Supplement: Supplementary Information [file lsa2017174x1.pdf]

Supplementary Information

Multispectral light scattering endoscopic imaging of esophageal precancer

Le Qiu[1], Ram Chuttani[2], Douglas K. Pleskow[2], Vladimir Turzhitsky[1], Umar Khan[1], Yuri N. Zakharov[1], Lei Zhang[1], Tyler M. Berzin[2], Eric U. Yee[3], Mandeep S. Sawhney[2], Yunping Li[4], Edward Vitkin[1], Jeffrey D. Goldsmith[3], Irving Itzkan[1] and Lev T. Perelman[1,2,5*]

[1]Center for Advanced Biomedical Imaging and Phonics, Division of Gastroenterology, Department of Medicine, Beth Israel Deaconess Medical Center, Harvard University, Boston, MA 02215, USA

[2]Division of Gastroenterology,
Department of Medicine, Beth Israel Deaconess Medical Center, Harvard University, Boston, MA 02215, USA

[3]Department of Pathology, Beth Israel Deaconess Medical Center, Harvard University, Boston, MA 02215, USA

[4]4Department of Anesthesia, Critical Care and Pain Medicine, Beth Israel Deaconess Medical Center, Harvard University, Boston, MA 02215, USA

[5]Biological and Biomedical Sciences Program
Harvard University, Boston, Massachusetts 02215 USA

*Corresponding author. E-mail: lperelman@fas.harvard.edu (L.T.P.)

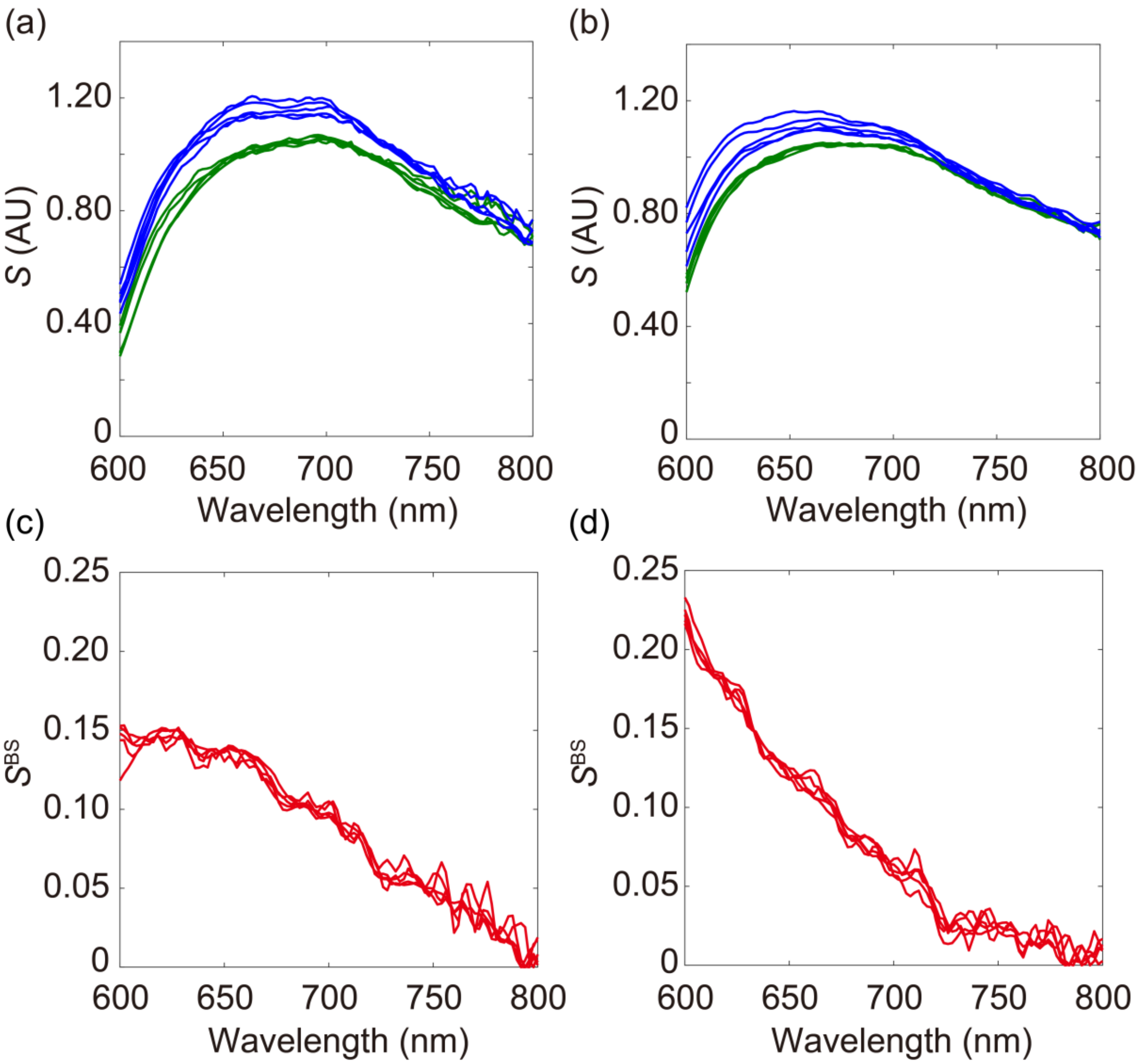

Figure S1. Typical spectra collected in the Barrett's esophagus. The parallel (blue) and perpendicularly (green) polarized reflectance spectra for NDB (a) and HGD (b) from five different locations each and the backscattering spectra for the same NDB (c) and HGD (d) locations.

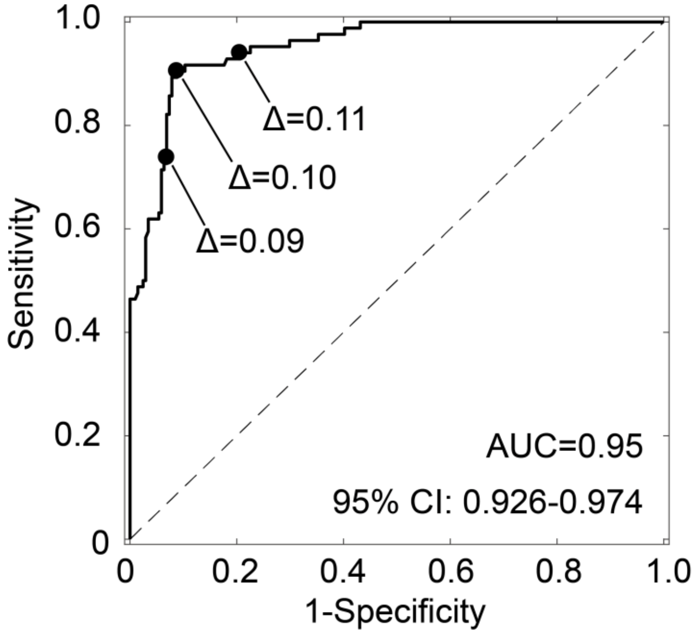

Figure S2. Receiver operating characteristic (ROC) analyses. ROC curve shows the performance of the LSS endoscopic imaging in identifying dysplastic sites in subjects with BE by comparing the LSS results with the definitive histopathology findings for all values of Δ in our data set. The area under the ROC curve (AUC) is 0.95 (95% confidence interval: 0.926-0.974, p<0.001). Diagnostic cut-offs $\Delta = 0.1$ along with $\Delta = 0.09$ and $\Delta = 0.11$ are shown for illustration purposes.

Movie S1. Tracking of locations suspicious for HGD. Three locations suspicious for HGD are marked with red, yellow, and violet solid circles. Four to seven trackable features per frame are marked with green triangles.
